# Analyzed PD-L1-positive subpopulations by dual-labeling TSA-IF-FISH predicts immunotherapy efficacy in advanced lung cancer

**DOI:** 10.1016/j.isci.2025.114357

**Published:** 2025-12-06

**Authors:** Lin Chen, Zhonglin Yang, Yue Lu, Shan Li, Dongjiang Tang, Lei Zhang

**Affiliations:** 1 MOE International Joint Research Laboratory on Synthetic Biology and Medicines, School of Biology and Biological Engineering, South China University of Technology, Guangzhou 510640, P.R. China; 2 Zhuhai Sanmed Biotech Ltd., 266 Tongchang Road, Zhuhai 519060, P.R. China

**Keywords:** health sciences

## Abstract

The evaluation of predictive biomarkers in advanced lung cancer requires methods that can comprehensively profile rare cell populations. We developed a dual-labeling assay integrating tyramide signal amplification immunofluorescence (TSA-IF) with fluorescence *in situ* hybridization (FISH) to concurrently detect protein expression and chromosomal aberrations. This approach was used to analyze PD-L1-positive circulating tumor cells (CTCs), circulating tumor endothelial cells (CTECs), and white blood cells (WBCs) from multiple biofluid types. Our assay improved signal integrity and revealed distinct clinical associations: specific PD-L1^+^ CTC phenotypes were linked to metastasis and correlated with improved immunotherapy response, whereas PD-L1^+^ CTECs were associated with treatment resistance and serum tumor markers. Furthermore, PD-L1^+^ WBC levels were strongly correlated with C-reactive protein, connecting them to systemic inflammation. This integrated liquid biopsy strategy enables a multifaceted view of the tumor microenvironment and host immune status, presenting a conceptual advance for monitoring treatment efficacy and inflammatory activity in advanced lung cancer.

## Highlights

• PD-L1^+^/Vimentin CTCs are correlate with immunotherapy response
• PD-L1^+^ CTECs are associated with immunotherapy resistance
• PD-L1^+^ WBCs correlate with inflammatory response

## Graphical abstract

**Figure:**
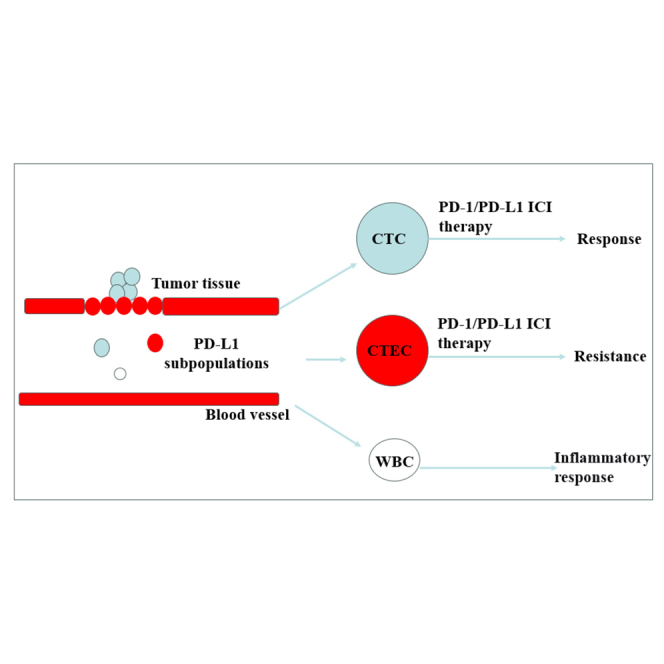

## Introduction

Circulating tumor cells (CTCs) have emerged as key components in liquid biopsy, offering insights into tumor progression,^1^ immune response,^2^ and treatment efficacy.^3^ CTC were detached from primary tumor and entered bloodstream,^4^ showed potential in monitoring disease progression^5^ and predicting treatment response.^6^^,^^7^ Previous research showed CTC exhibited significant heterogeneity in biomarker expression,^8^ including the presence of epithelial biomarker (cytokeratin [CK], epithelial cell adhesion molecule [EpCAM]) and mesenchymal markers (vimentin),^9^ as well as immune checkpoint proteins such as PD-L1.^10^ CTCs expressing programmed death ligand 1 (PD-L1^+^ CTC) are implicated in immune evasion and may serve as predictive biomarkers for response to immune checkpoint inhibitors.^11^ For instance, CK-positive and PD-L1-positive (CK^+^/PD-L1^+^) CTCs have been identified in various biofluids from advanced lung cancer patients and have shown significant associations with objective response rate (ORR) to PD-1/PD-L1 inhibitors.^12^ Furthermore, some subtypes of CTCs, such as PD-L1^+^ vimentin CTCs (PD-L1^+^/VIM^+^ CTC) was associated to worse PFS and OS,^13^^,^^14^ but there are no studies on the relationship of PD-L1^+^/VIM^+^ CTC to PD-1/PD-L1 immunotherapy.

Similarly, circulating tumor endothelial cells (CTECs)—defined by aneuploidy and tumor vascular origin^15^—were identified that associated with metastatic disease and poor prognosis,^16^ and linked to diminished clinical benefits and shorter progression-free survival in non-small cell lung cancer patients undergoing anti-PD-1/PD-L1 therapy, suggesting an active role in mediating immune evasion.^17^ However, the differential roles and clinical relevance of PD-L1 expression CTCs, CTECs, and white blood cells (WBCs) remain poorly characterized, especially in the context of therapeutic resistance and inflammatory responses. Understanding these associations can help guide the development of more effective therapeutic strategies.^18^^,^^19^

A major challenge in dissecting these populations lies in their immunophenotypic overlap, particularly among small nuclear-sized cells, which complicates discrimination by conventional immunofluorescence (IF). While fluorescence *in situ* hybridization (FISH) can resolve such ambiguity through chromosomal analysis,^20^ standard IF-FISH workflows often compromise antigen integrity during denaturation.^21^^,^^22^ To circumvent this limitation, we implemented tyramide signal amplification (TSA)-based immunofluorescence (TSA-IF), which preserves epitope integrity under stringent FISH conditions by catalytically depositing fluorophores at tyrosine residues.^23^^,^^24^^,^^25^ This strategy enables simultaneous protein and genetic profiling at single-cell resolution.

Leveraging this integrated TSA-IF-FISH platform, we aim to characterize PD-L1^+^ CTCs, CTECs, and WBC subpopulations and assess their clinical relevance in advanced lung cancer patients undergoing immunotherapy. Our study seeks to clarify the relationship between these circulating cells and treatment efficacy, with the goal of informing more personalized therapeutic strategies.

## Results

### Optimization and application of TSA-IF in combined molecular profiling for CTC analysis

**Figure 1:**
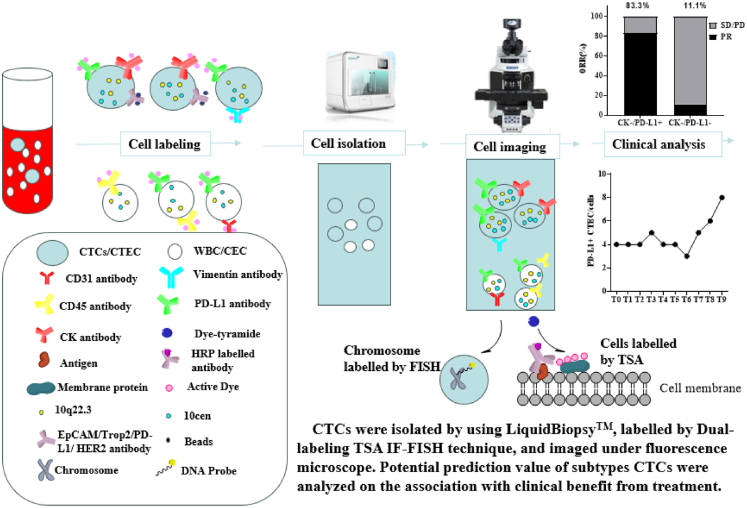
Schematic of the dual-labeling TSA IF-FISH for CTCs/CTEC and WBCs/CEC Subtype of CTCs, CEC, and WBCs were isolated by using LiqiudBiopsy™ system from 5 to 10 mL blood/PE/CSF and detected by dual-labeling TSA IF-FISH technique based on anti CK/PD-L1/Vimentin/CD31/CD45 antibodies and chromosome 10 probes to analyze the correlation to clinical benefit from treatment. CTCs, circulating tumor cells; WBC, white blood cell; RBC, red blood cell; CD45, leukocyte common antigen 45; PD-L1, programmed death-ligand 1; CK, cytokeratin; HRP, horseradish peroxidase; TSA, tyramide signal amplification; FISH, fluorescence *in situ* hybridization; CEC, circulating endothelial cells; CTECs, circulating tumor endothelial cells; PE, pleural effusion; CSF, cerebrospinal fluid

**Figure 2:**
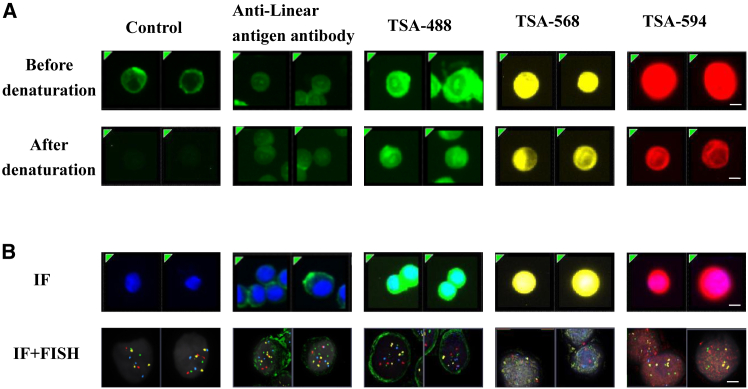
Construction of IF + FISH detection method by TSA IF + FISH detection system was constructed by using anti-linear antigen HER2 antibody and the TSA method before or after denaturation on SKBR3. Centromeric probes of chromosome 10q22.3 (yellow), 10cen (blue), and chromosome 3p22.1 (red) and 3q29 (green) were used for FISH detection. Results demonstrated that cells maintained a remarkable detection signal even after denaturation when using TSA-488 (test with HRP labeled anti HER2 antibody), TSA-568 (test with HRP labeled anti-EpCAM antibody), and TSA-594 (test with HRP labeled anti pan CK antibody) (A) Overcoming Antigen Degradation in IF-FISH Assays through Tyramide Signal Amplification (TSA). (B) Application of TSA-IF-FISH for Enhanced Detection of HER2, EpCAM, and CK and Chromosomal Abnormalities in Circulating Tumor Cells. Bars: 8 μm. HER2, Human epidermal growth factor receptor 2; EpCAM, Epithelial cell adhesion molecule.

Conventional FISH assays necessitate thermal denaturation (72°C–85°C) to unwind DNA duplexes for probe hybridization, which disrupts the conformational integrity of cell surface antigens and compromises subsequent IF detection. To address this technical incompatibility, we employed a TSA-based approach, utilizing fluorophore-conjugated tyramide substrates (488, 568, or 594 nm) that were covalently immobilized to cellular tyrosine residues, thereby bypassing antibody-antigen binding dependency (Figure 1). Strikingly, TSA-IF demonstrated robust fluorescence signals regardless of denaturation sequence—whether performed pre- or post-fluorophore deposition—while conventional antibody-based IF exhibited complete signal loss after denaturation. Antibodies targeting linear epitopes retained partial binding capacity to denatured antigens but produced markedly attenuated signals (Figure 2A). These findings validate the superiority of TSA in preserving antigen detection fidelity under denaturation stress. The covalent anchoring mechanism of TSA effectively decouples fluorophore stability from antigen structural integrity, thereby resolving the long-standing incompatibility between DNA denaturation and antigen preservation in sequential IF-FISH assays. This methodological advance enables reliable co-detection of genetic and protein biomarkers in combined workflows, with TSA-IF emerging as the optimal strategy for integrated molecular profiling.

To further validate the practical utility of this approach, we applied TSA detection technology in combination with IF and FISH to analyze HER2 protein, EpCAM protein, and CK protein expression and chromosomal abnormalities in CTCs. Using TSA-488 to stain HER2 antigen, TSA-568 to detected EpCAM antigen, and TSA-594 label CK antigen in SKBR3 cells, followed by detection of specific probes for chromosomes 10, we demonstrated that TSA significantly enhanced IF detection signals compared to traditional methods and specialized antibody techniques. Specifically, TSA-IF resulted in a marked increase in signal intensity compared to control and antibodies targeting linear antigens. Moreover, the combined IF and FISH results revealed distinct membrane staining signals for protein along with chromosomal hybridization signals in CTCs (Figure 2B). This integrated approach not only overcomes the limitations of antigen destruction during FISH but also enhances the sensitivity of detection for both protein expression and chromosomal abnormalities. Our findings suggest that the integration of TSA detection technology with IF and FISH provides a robust platform for the identification of protein and chromosomal abnormalities in CTCs, potentially advancing diagnostic capabilities in oncology. Further studies are warranted to explore the clinical implications and applications of this method in cancer diagnosis and monitoring.

### CK^−^/PD-L1^+^ CTCs as a potential biomarker for predicting immunotherapy efficacy in advanced lung cancer

**Figure 3:**
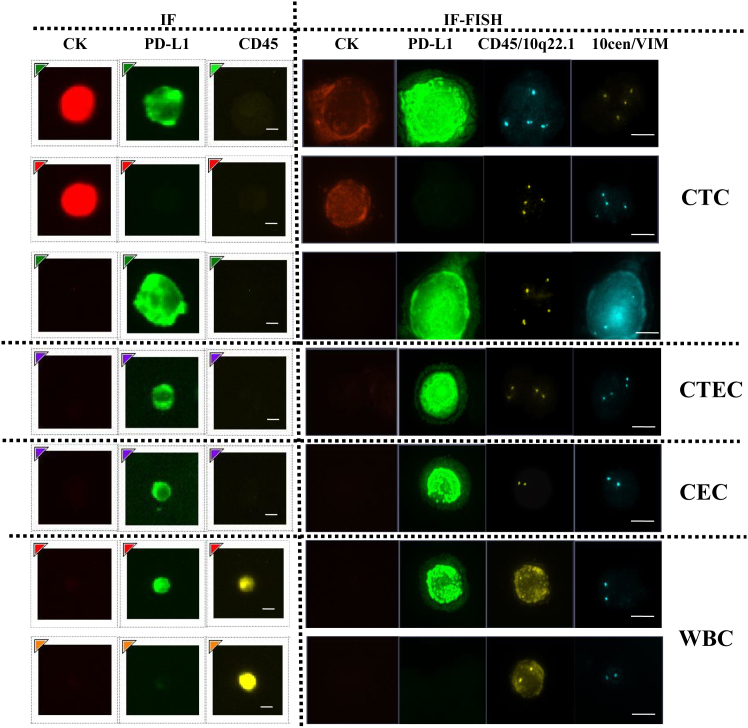
Evaluation of subtype of CTCs and WBCs by using IF and IF-FISH method respectively Clinical samples were collected and isolated by using LiquidBiopsy™ system, and detected by using IF and IF-FISH respectively based on anti CK/PD-L1/Vimentin/CD45 antibodies and chromosome 10 probes. Centromeric probes of chromosome 10q22.3 (yellow) and 10cen (blue) were used for FISH detection. Subtypes of CTCs and WBCs were imaged by using Leica DM6000B (IF test) and Bioview microscope (IF-FISH test). Results showed that each subtypes CTCs and WBCs could be classified clearly (A–G) (A–C) CTCs subtypes with CK+/PD-L1+/VIM^−^/CD45-, CK+/PD-L1-/VIM^−^/CD45-, and CK^−^/PD-L1^+^/VIM^+^/CD45^-^, respectively; (D) CTEC with CK^−^/PD-L1^+^/VIM^−^/CD45^-^; (E) CEC with CK^−^/PD-L1^+^/VIM^−^/CD45^-^; (F–G) WBCs subtypes with CK^−^/PD-L1^+^/CD45^+^ and CK^−^/PD-L1^-^/CD45^+^ respectively. Bars: 8 μm. VIM, vimentin.

**Figure 4:**
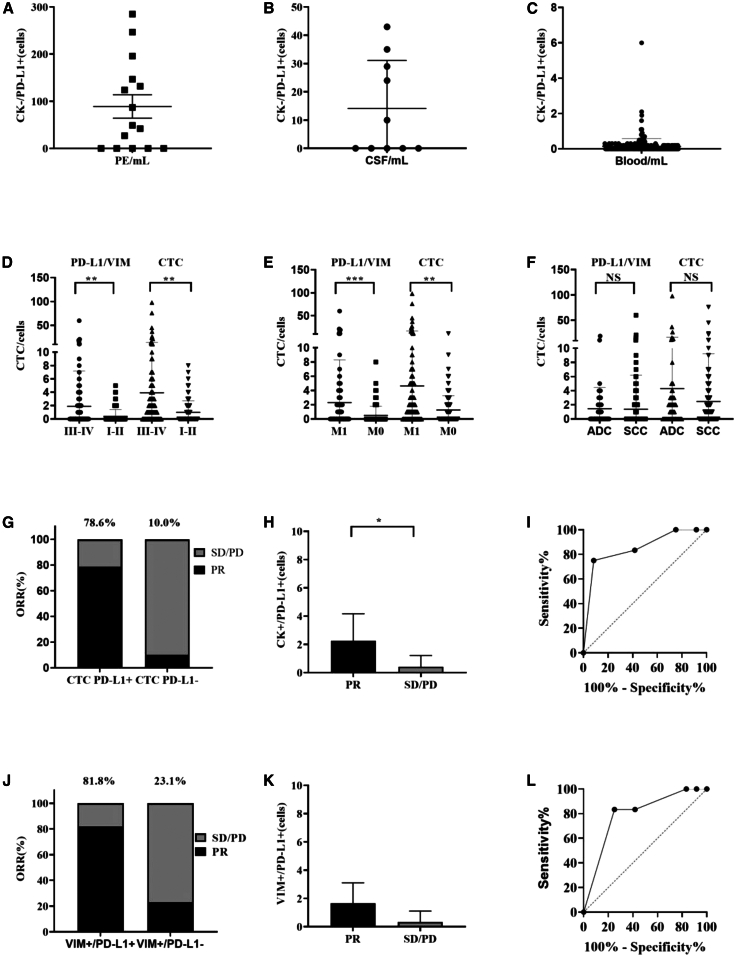
Clinical prognostics values of CK^−^/PD-L1^+^ CTCs in lung cancer CK^−^/PD-L1^+^/VIM+ cells were assessed by IF-FISH on 5–10 mL pleural effusions (PE) (89.1 ± 95.8 cells/mL), cerebrospinal fluid (CSF) (14.1 ± 17.0 cells/mL), and blood samples (0.14 ± 0.44 cells/mL) at lung cancer patients, respectively. Quantity of CK^−^/PD-L1^+^/VIM+ cells was analyzed on Student’s *t* test, which showed a significance to stage (*p* = 0.0073, 95% CI -2.587 to −0.4070) and metastasis (*p* = 0.001, 95% CI -2.842 to −0.7325), but not to cancer subtype (*p* = 0.9128, 95% CI -1.276 to 1.141) at 278 lung cancer blood samples. CK^−^/PD-L1^+^/VIM+ cells was also evaluated at baseline (before PD-1/PD-L1 immunotherapy) in 24 patients and analyzed on the association with objective response rate (ORR) after 2 cycles of PD-1/PD-L1 immunotherapy. CK^+^/PD-L1^+^ CTCs were correlated with higher ORR significantly (*p* = 0.0056 < 0.05) and acquired high sensitivity (75.0%) and specificity (91.7%) when the cutoff at > 0.5 with AUC 0.8507 (*p* = 0.0035 < 0.05; 95% CI 0.6908 to 1.000) based on ROC curve analysis. Moreover, CK-/PD-L1+/VIM+ cells were also correlated with higher ORR significantly (*p* = 0.0098 < 0.05) and acquired high sensitivity (83.3%) and specificity (75.0%) when the cutoff at >0.5 with AUC 0.7917 (*p* = 0.0153 < 0.05; 95% CI 0.6032 to 0.9832) (A–F) (A–C): Evaluation of CK^−^/PD-L1^+^ CTCs at PE, CSF, and blood samples of lung cancer, respectively; (D–F): Association of CK^−^/PD-L1^+^ CTCs to stage, and metastasis, and cancer subtype of lung cancer in blood samples, respectively. (G–L) (G–H) Comparative analysis of ORR between patients with CTC PD-L1 status and quantities respectively; (I): ROC curve analysis of the relationship of CTC PD-L1 with ORR; (J–K): Comparative analysis of ORR between patients with CK^−^/PD-L1+/VIM^+^ status and quantities; (L): ROC curve analysis of the relationship of CK^−^/PD-L1+/VIM^+^ cells with ORR. Significant relevance was analyzed by using students’ *t* test at *p* ≤ 0.05 (∗), *p* ≤ 0.01 (∗∗), and *p* ≤ 0.001 (∗∗∗). Data are represented as mean ± SEM. ADC, Adenocarcinoma; SCC, Squamous cell carcinoma; NS, None significance; PR, Partial response; SD, Stable disease; PD, Progressive disease; ORR, Objective response rate; AUC, Area under the curve; CI, confidence interval. ROC, Receiver operating characteristic.

CTCs exhibiting heterogeneous biomarker expression, including CK-/EpCAM-phenotypes with positivity for PD-L1^26^ or vimentin,^27^ were identified and validated in previously study. In the study, distinct IF signals (reflecting protein expression) and FISH signals (confirming chromosomal aberrations) were observed in CK^+^/PD-L1^+^ (Figure 3A), CK^+^/PD-L1^-^ (Figure 3B), and CK^−^/PD-L1^+^ (Figure 3C) subpopulations, which were different from CEC subtype (CK-/PD-L1+/CD45-/VIM-) (Figures 3D and 3E) and WBC subtype, CK^−^/PD-L1^+^/CD45^+^(Figure 3F), and CK^−^/PD-L1/CD45^+^ (Figure 3G). CK^−^/PD-L1^+^ CTCs were detected in 66.7% (10/15) of PE with the quantity of 89.1 ± 95.8 cells/ml (Figure 4A), 50% (5/10) of CSF samples with the quantity of 14.1 ± 17.0 cells/mL (Figure 4B), and 32.3% (90/278) of blood samples with the quantity of 0.14 ± 0.44 cells/mL (Figure 4C), from advanced lung cancer patients, highlighting their widespread distribution across biofluids. Furthermore, the quantity of CK^−^/PD-L1^+^ CTCs was significant to stage (*p* = 0.0073, 95% CI –2.587 to −0.4070) (Figure 4D) and metastasis (*p* = 0.001, 95% CI –2.842 to −0.7325) (Figure 4E), but not to cancer subtype (*p* = 0.9128, 95% CI –1.276 to 1.141) at lung cancer blood samples (Figure 4F).

In a cohort of 24 patients receiving PD-1/PD-L1 immunotherapy, CTCs was positive in 62.5% (15/24) patients. In the 14 CK^+^/PD-L1^+^ patients (14/24, 58.3%), 11 (11/14, 78.6%) individuals acquired PR, compared to PD-L1-negative patients, where only one case of PR (1/10, 10.0%) was observed alongside eight cases of stable disease (SD) and one progressive disease (PD) (Figure 4G). Additionally, in the 11 CK^-^/PD-L1^+^/VIM^+^ positive patients, nine patients achieved PR (9/11, 81.8%), while in the 13 CK^-^/PD-L1^+^/VIM^+^ negative patients, only 3 patients acquired PR (3/13, 23.1%) (Figure 4J). The overall concordance between CK^−^/PD-L1^+^/VIM^+^ cells detection and ORR was 79.2% (19/24), with chi-square analysis confirming a statistically significant association (*p* = 0.005) (Table 1). Moreover, not only the quantity of CK PD-L1 (*p* = 0.0056, Figure 4H) but also CK^−^/PD-L1^+^/VIM+ was significant difference in PR and SD/PD with *p* value 0.0142 (Figure 4K). ROC curve analysis showed that not only CTC PD-L1 would be reliable in prediction benefit from PD-1/PD-L1 immunotherapy with AUC = 0.8507 > 0.8 (cutoff at >0 with AUC 0.8507; *p* = 0.0035 < 0.05; 95% CI 0.6908 to 1.000; sensitivity 75.0% and specificity 91.7%; Figure 4I), but also CK^−^/PD-L1^+^/VIM^+^ (cutoff at >0.5 with AUC 0.7917 (*p* = 0.0153 < 0.05; 95% CI 0.6032 to 0.9832; sensitivity 83.3% and specificity 75.0%; Figure 4L) had potential act as a valuable clinical biomarker in immunotherapy. These findings underscore the clinical relevance of PD-L1^+^ CTC (no matter CK^+^ or VIM^+^) as a potential biomarker for predicting immunotherapy efficacy in advanced lung cancer.

**Table 1:** Assessment of clinical and laboratory variables in partial response (PR) and stable disease/progressive disease (SD/PD) at baseline (T0)

	PR	SD/PD	*P*
Age	69.50 ± 6.260	64.33 ± 8.616	0.628
Gender			0.083
Male	12	9	NA
Female	0	3	NA
Cancer subtype			1.000
Adenocarcinoma	5	5	NA
Squamous cell carcinoma	7	7	NA
Tissue PD-L1			0.688
<1%	5	6	NA
>1%	7	6	NA
PD-1 or PD-L1 therapy			0.284
PD-1	9	11	NA
PD-L1	3	1	NA
CTC CK			0.216
Positive	6	3	NA
Negative	6	9	NA
CTC PD-L1			0.001∗∗∗
Positive	11	3	NA
Negative	1	9	NA
CK-/PD-L1^+^/VIM+			0.005∗∗
Positive	9	2	NA
Negative	3	10	NA
CTEC			0.09
Positive	2	7	NA
Negative	10	5	NA
Stage			0.105
I-III	9	7	NA
IV	3	5	NA
CEA	11.38 (1.91, 88.54)	13.63 (2.47, 217.09)	0.436
CA125	12.21 (5.93, 689.00)	12.79 (15.10, 393.00)	0.840
CYFRA21-1	12.29 (2.60, 48.40)	12.71 (3.15, 18.90)	0.885
NSE	11.58 (5.23–3454.00)	13.43 (15.20–63.00)	0.525
CRP	11.25 (4.80–84.70)	13.75 (6.43–37.90)	0.386

Significant relevance was analyzed by using students’ *t* test at *p* ≤ 0.05 (∗), *p* ≤ 0.01 (∗∗), and *p* ≤ 0.001 (∗∗∗) on 24 patients.

CA125, Carbohydrate Antigen 125; CEA, Carcinoembryonic antigen; CK, Cytokeratin; CTCs, Circulating tumor cells; CTECs, Circulating tumor endothelial cells; CRP, C-reactive protein; CYFRA21-1, Cytokeratin fragment 21-1; PD-1, Programmed death 1; PD-L1, Programmed death ligand 1; PR, Partial response; SD, Stable disease; PD, Progressive disease. Pleural effusion; ROC, receiver operating characteristic; NSE, neuron-specific enolase.

### Differential dynamics and correlation analysis of PD-L1^+^ CTEC_S_ and WBCs in lung cancer: Insights from dual-labeling assays

**Figure 5:**
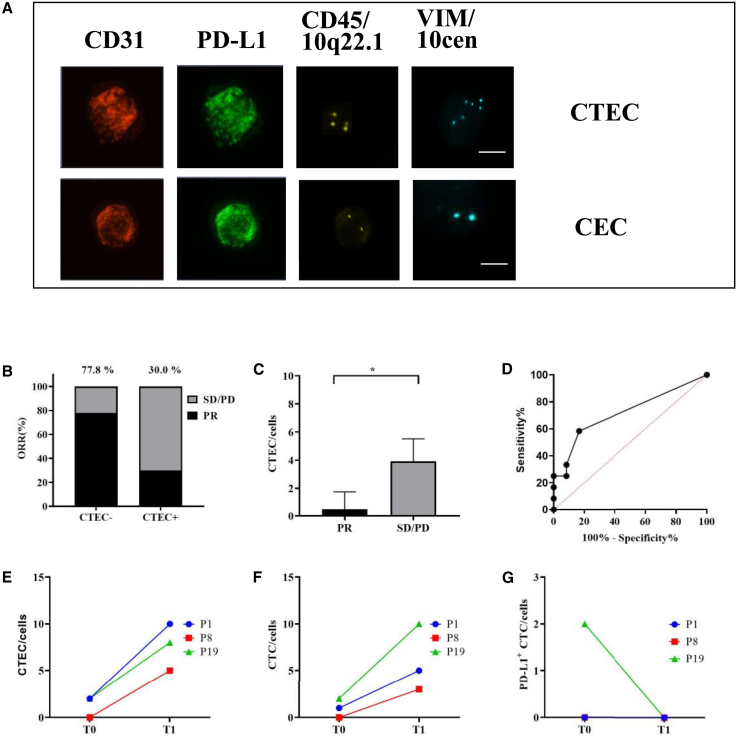
Association of CTEC to clinical benefit from immunotherapy CTEC (with CD31+/PD-L1+/CD45-/VIM-) was detected in 9 patients and analyzed on the association to clinical benefit from PD-1/PD-L1 immunotherapy at T0 (baseline) by Student’s *t* test. Additionally, 21 patients with PR/SD were collected blood for CTC evaluation and also for CTEC analysis at T1 (After 2 cycles of immunotherapy). CTECs were correlated with lower ORR significantly (77.8% vs. 30%; *p* = 0.0487 < 0.05), but with low sensitivity (58.33%) and specificity (83.33%) when the cutoff at >1 with AUC 0.7188 (*p* = 0.069 < 0.05; 95% confidence interval 0.5085 to 0.9290) at T0. Notably, longitudinal monitoring revealed that all three patients who developed PD exhibited increased counts of both CTECs and CTCs at T1 compared to baseline. In contrast, among the 18 patients with PR/SD, CTEC and CTC levels either decreased or remained stable (A–G) (A) Characterization of CTEC and CEC; (B–C) Correlation between quantity of CTEC and ORR at T0; (D) ROC curve analysis of the relationship of CTCE with ORR at T0; (E–G) Dynamic monitoring of CTEC, CTC, and PD-L1+ CTC, respectively. Significant relevance was analyzed by using students’ *t* test at *p* ≤ 0.05 (∗), *p* ≤ 0.01 (∗∗), and *p* ≤ 0.001 (∗∗∗) at 24 patients. Data are represented as mean ± SEM. Bars: 8 μm. Abbreviations: CD31, Platelet endothelial cell adhesion molecule-1, PECAM-1.

During the investigation, we observed that a subset of patients, particularly those with SD or PD, exhibited detectable levels of CTECs (Figures 3D and 5A). These CTECs (defined as CD31^+^/PD-L1^+^/CD45^-^/VIM^−^) were phenotypically distinct from both PD-L1^+^/VIM^+^ cells (Figures 3C and 5A) and normal CECs (Figures 3E and 5A). At baseline (T0), CTECs were detected in nine patients and significantly associated with reduced clinical benefit from anti-PD-1/PD-L1 therapy. The ORR was markedly lower in CTEC-positive patients compared to CTEC-negative patients (30% vs. 77.8%). Furthermore, patients with partial response (PR) had significantly fewer CTECs than those with SD or PD (*p* = 0.0478), though using a cutoff of >1 CTEC at T0 for predicting treatment response showed limited sensitivity (58.33%) and specificity (83.33%), with an AUC of 0.7188 (*p* = 0.069; 95% CI, 0.5085–0.9290). Longitudinal analysis revealed that all three patients who was PD showed increased counts of both CTECs (Figure 5E) and CTCs (Figure 5F) at T1 relative to baseline, while PD-L1 CTCs quantity was decreased or stable (Figure 5G).

**Figure 6:**
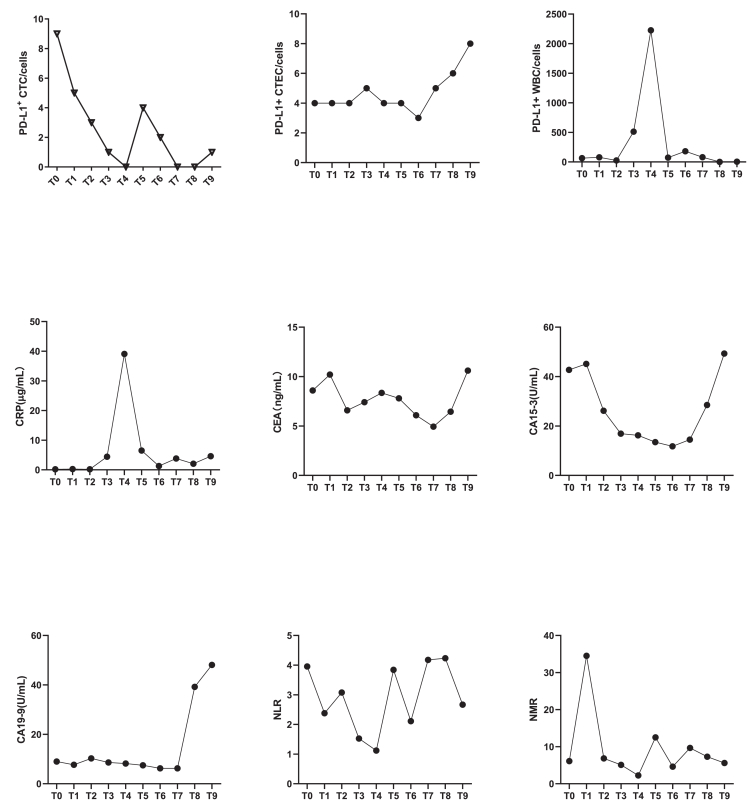
A case report of dynamic monitoring in biomarkers (P11) An advance lung cancer (tissue PD-L1 = 10%) patient was accepted chemotherapy with immunotherapy (paclitaxel protein-bound with pembrolizumab) and followed up for 15 months. PD-L1^+^ CTC was decreased during therapy constantly, while PD-L1^+^ CTEC was stable during T0 to T6, and increased at T7 with progression disease. PD-L1+ WBCs was associated to the CRP (Spearman’s rho = 0.875, *p* < 0.001) (A–I) (A) Dynamic monitoring of CTC during therapy; (B–C) Dynamic changes of the quantity of PD-L1^+^ CTEC and PD-L1+ WBCs during therapy respectively; (D–G) Dynamic changes of CRP, CEA, CA15-3, and CA19-9 during therapy, respectively; (H–I) Dynamic changes of NLR and NMR respectively. CRP, C-reactive protein; CEA, Carcinoembryonic antigen; CA15-3: Carbohydrate Antigen15-3; CA19-9, Carbohydrate antigen 19-9; NLR, Neutrophil-to-Lymphocyte Ratio; NMR, Neutrophil-to-Monocyte Ratio.

Additionally, the PD-L1^+^ CTECs count originating from tumor cells exhibited minimal variation across treatment phases from T0 to T6, whereas CTC PD-L1 levels decreased significantly during therapy in P11 (Figures 6A and 6B). This pattern suggests inherent resistance to pharmacological agents and immune-mediated clearance. Notably, an increase in PD-L1^+^ CTECs (Figure 6B), CEA (Figure 6E), CA15-3 (Figure 6F), and CA19-9 (Figure 6G) was observed at T7 during disease progression and further investigation revealed that significant positive associations between PD-L1^+^ CTECs and CEA (Spearman’s rho = 0.654, *p* < 0.01), CA15-3 (Spearman’s rho = 0.587, *p* < 0.01), and CA19-9 (Spearman’s rho = 0.456, *p* < 0.05) (Table 2). Additionally, PD-L1^+^ WBCs peaked at T4 during an inflammatory phase and significantly correlated with CRP levels (Spearman’s rho = 0.875, *p* < 0.001) (Table 2). These findings indicate that PD-L1^+^ CTECs may serve as a potential biomarker for tumor burden and drug resistance, while PD-L1^+^ WBCs may be associated with inflammatory responses.

**Table 2:** Correlation analysis of PD-L1^+^ CTEC to tumor biomarker and PD-L1^+^ WBC to CRP

Variable 1	Variable 2	Spearman’s rho	*p*-value
PD-L1^+^ CTEC	CEA	0.654	<0.01∗∗
PD-L1^+^ CTEC	CA153	0.587	<0.01∗∗
PD-L1^+^ CTEC	CA199	0.456	<0.05∗
PD-L1^+^ WBC	CRP	0.875	<0.001∗∗∗

Correlation was analyzed by using Spearman’s rho with significant relevance at *p* ≤ 0.05 (∗), *p* ≤ 0.01 (∗∗), and *p* ≤ 0.001 (∗∗∗).

WBC, white blood cell.

## Discussion

This study provides a comprehensive investigation into dynamics and clinical relevance of PD-L1 expression CTCs, CTEC, and WBCs based on LiquidBiopsy system, which achieved high accuracy in the validation cohort (Tables S1 and S2) and specific detection in clinical samples (Table S3). Using dual-labeling IF and FISH assays (validated in Figures S1 and S2), we identified distinct subpopulations and revealed their differential dynamics during therapeutic. A key finding is the identification of PD-L1^+^ CTCs (including CK+/PD-L1+ and VIM+/PD-L1+ phenotypes) were significantly associated with ORR, identifying them as a potential predictive biomarker that appears to function independently of tPD-L1 status, with which no association was fund (Tables S4 and S5). Additionally, PD-L1^+^ CTECs emerged as a potential indicator of tumor burden and drug resistance, while PD-L1^+^ WBCs were linked to inflammatory responses.

Our findings align with recent studies highlighting the importance of CTCs in predicting treatment outcomes,^28^^,^^29^^,^^30^ but extend this knowledge by focusing on specific biomarker phenotypes (CK^−^/PD-L1^+^/VIM^+^) and their clinical relevance in advanced lung cancer. The high concordance between PD-L1^+^ CTC detection and ORR underscores their potential utility in guiding personalized treatment strategies. This is particularly important given the growing emphasis on precision medicine in cancer therapy, where biomarkers can help tailor treatments to individual patients, potentially improving outcomes and reducing unnecessary side effects.^31^

The application of TSA technology in our IF-FISH assays significantly enhanced detection sensitivity and reliability, enabling more accurate molecular profiling of these rare cell populations and overcoming limitations of traditional methods. This technological advancement is crucial for the reliable identification and characterization of CTCs, CTECs, and WBCs, which can vary significantly in their biomarker expression and functional properties. By improving detection methods, we can better understand the heterogeneity of these cells and their roles in disease progression and treatment response.

In summary, our study provides preliminary evidence on the dynamics and clinical relevance of PD-L1^+^ CTCs, PD-L1^+^ CTECs, and PD-L1^+^ WBCs in advanced lung cancer. The association between PD-L1^+^ CTCs/CTECs and treatment response supports their potential role as exploratory immunotherapy efficacy. Future validation in larger, prospective cohorts is required to confirm these findings and assess their generalizability across diverse cancer types. Addressing the current limitations through integrated molecular analyses and advanced technologies will be essential to clarify the biological functions of these circulating cells and their possible utility in clinical practice.

### Limitations of the study

We acknowledge the limitation of our study, including the small cohort of patients receiving PD-1/PD-L1 therapy, its focus on late-stage lung cancer, and the lack of mechanistic insights into the roles of PD-L1^+^ CTECs to therapeutic resistance and PD-L1^+^ WBCs to inflammatory. Future studies should involve larger, multi-center, longitudinal cohorts across diverse malignancies; combine single-cell sequencing and advanced imaging to decipher cellular heterogeneity and origin; and integrate TSA-based liquid biopsy with next-generation sequencing to realize adaptive precision therapy. Furthermore, due to the limited sample size, we did not perform an independent prognostic analysis based exclusively on FISH-confirmed cell subsets. Future studies with larger prospective cohorts are warranted to explore the potential superior prognostic or predictive value of aneuploidy-confirmed CTCs and CTECs over immunophenotyping alone.

## Resource availability

### Lead contact

Further information and requests for resources and reagents should be directed to and will be fulfilled by the lead contact, Lin Chen (chen.lin@sanmedbio.com).

### Materials availability

This study did not generate new unique reagents.

### Data and code availability

• All data in this paper will be shared by the lead contact upon request.
• This paper does not report original code.
• Any additional information required to reanalyze the data reported in this paper is available from the lead contact upon request.

## Acknowledgments

This study was partly funded by the Guangdong Provincial Medical Products Administration (No. 2024ZDZ01).

## Author contributions

S.L., T.D., and Z.L. conceptualized, designed, and supervised the study and reviewed and critically revised the manuscript; L.C. designed and performed most of the experiments; acquired, analyzed and interpreted data; and drafted the manuscript. Z.Y. and Y.L. provided technical support.

## Declaration of interests

The authors declare that they have no competing interests.

## STAR★Methods

### Key resources table

**Table:** 

REAGENT or RESOURCE	SOURCE	IDENTIFIER
**Reagents**		

Telomerase-specific probes TSA-488, TSA-568, and TSA-594	Mao et al.^25^	NA
Ficoll-Paque	Solarbio	P8610
4% paraformaldehyde	Solarbio	P1110
Streptavidin-coated particle beads	BD Biosciences	#557812

**Antibodies**		

Alexa Fluor 488-conjugated anti-HRP secondary antibody	Jackson ImmunoResearch	#123-545-021
CK-iFluor 647	Zhuhai Sanmed Biotech Ltd.	NA
PD-L1-iFluor 488	Zhuhai Sanmed Biotech Ltd.	NA
CD45-iFluor 568	Zhuhai Sanmed Biotech Ltd.	NA
Alexa Fluor 488–conjugated anti-HER2 antibody	Santa Cruz Biotechnology	sc-7301 AF488
HRP-conjugated antibodies against HER2, CK, PD-L1, CD45, Vimentin, CD31 capture cocktail kit^32^	Zhuhai Sanmed Biotech Ltd.	NA
biotinylated capture antibodies (against HER2, Trop2, EpCAM, and PD-L1)	Zhuhai Sanmed Biotech Ltd.	NA

**Probes**		

Centromeric probes for chromosome 10q22.3 (Gold spectrum),10cen (Aqua spectrum) and chromosome 3p22.1 (Red spectrum) and 3q29 (Green spectrum)^33^	Zhuhai Sanmed Biotech Ltd.	NA

**Cell lines**		

SKBR3, NCI-H820, MCF-7, A549	ATCC	NA

### Experimental model and study participant details

#### Cell lines

All cell lines used in this study (SKBR3, NCI-H820, MCF-7, A549) were bought from ATCC, and authenticated by short tandem repeat profiling. All cell lines were confirmed to be free of mycoplasma contamination by PCR testing prior to experimentation, and maintained in Dulbecco’s modified Eagle’s medium (DMEM) supplemented with 10% fetal bovine serum (FBS, Gibco, Grand Island, NY, USA) at 37°C in a humidified atmosphere containing 5% CO_2_.

#### Clinical simples

• **Sample Size:** This study utilized a total of 327 samples from advanced lung cancer patients to investigate PD-L1+/Vimentin+ circulating tumor cells (CTCs) and PD-L1+ circulating tumor endothelial cells (CTECs). The cohort consisted of **15 pleural effusion samples**, **10 cerebrospinal fluid (CSF) samples** from patients with brain metastases, and **278 peripheral blood samples** from a broader cohort of advanced lung cancer patients. Additionally, a separate subset of **24 blood samples** from patients who received PD-1/PD-L1 immunotherapy was analyzed specifically for PD-L1+ CTCs and PD-L1+ CTECs. The sample sizes for the primary cohort were determined based on sample availability during the study period, while the immunotherapy subgroup included all eligible patients meeting the clinical criteria within our recruitment window.
• **Allocation to Experimental Groups:** Subjects and samples were allocated into distinct experimental groups based on **their sample type and clinical disease status**:
  1. The **Pleural Effusion Group** (*n* = 15): comprised of lung cancer patients with malignant pleural effusions.
  2. The **CSF Group** (*n* = 10): comprised exclusively of lung cancer patients with confirmed brain metastases.
  3. The **Advanced Lung Cancer Blood Cohort** (*n* = 278): included a general population of advanced lung cancer patients used for the primary CTC analysis.
  4. The **Immunotherapy-Treated Subgroup** (*n* = 24): This group was defined by a specific clinical intervention (receipt of PD-1/PD-L1 inhibitors) and was analyzed separately for correlative studies with treatment.

### Method details

#### Clinical specimen collection and storage

The specimens-including peripheral blood, Pleural effusion (PE), and Cerebrospinal fluid (CSF)-were processed on microfluidic chips at Zhuhai Sanmed Biotech Ltd. After on-chip washing, cells were cytospun (Cytospin 4, Thermo Scientific) onto a slide and detected by using dual labeling TSA IF-FISH technique to analyze the association of subtype of CTCs and WBCs to clinical benefit (Figure 1), or resuspended in freezing medium (90% FBS +10% DMSO) and stored at −80°C until further analysis.

#### Development of IF & FISH detection method

Conventional IF combined with FISH is technically challenging because the high-temperature denaturation step (72°C–85°C) required for FISH disrupts protein conformation, thereby preventing antibody binding to conformational epitopes. To overcome this limitation, we adopted an approach that relies on antibodies recognizing linear epitopes and integrated TSA. SKBR3 cells were first incubated with a horseradish peroxidase (HRP)-conjugated anti-HER2 antibody after Endogenous peroxidase blocked by using 1% H_2_O_2_ for 15 min at 37°C. In parallel, to validate epitope linearity, a subset of cells was treated with an Alexa Fluor 488–conjugated anti-HER2 antibody (Santa Cruz Biotechnology, sc-7301 AF488). After extensive washing, cells were sequentially exposed to an anti-HRP AF488 antibody and TSA-488 for 30 min at room temperature. Cells were then cytospun onto glass slides and subjected to standard FISH protocols, including a stringent wash in 2× SSC buffer at 74°C for 2 min to denature cellular antigens. Finally, fluorescence signals were acquired by microscopy.

For integrated IF and FISH analyses, SKBR3 cells were first incubated with HRP-conjugated anti-HER2 antibody; an Alexa Fluor 488-conjugated anti-HER2 antibody was applied in parallel to verify recognition of linear epitopes. After three PBS washes, cells were treated with anti-HRP AF488 antibody and TSA-488 (anti EpCAM antibody was used for TSA-568 testing, and anti-pan CK antibody was used for TSA-594 testing) for 30 min at room temperature. Cells were cytospun onto glass slides and imaged on a Leica fluorescence microscope using a 10× air objective to record IF signals. Slides were then subjected to standard FISH processing: 2× SSC at 74°C for 2 min for antigen denaturation, 0.5% pepsin digestion for 5 min at 37°C, and sequential dehydration in 70%, 85%, and 100% ethanol (1 min each). A probe hybridization mixture targeting chromosomes 3 and 10 was applied and incubated for 24 h at 39°C according to the manufacturer’s protocol. Post-hybridization stringency washes were performed with 0.4× SSC at 74°C for 2 min followed by 2× SSC at 37°C for 3 min. Nuclei were counterstained with DAPI, and FISH signals were acquired on a Bioview fluorescence microscope (Bioview, Israel) equipped with a 60× oil-immersion objective.

#### CTC isolation, detection, and characterization

CTCs were prospectively isolated from peripheral blood (Data S1A), pleural/peritoneal effusion (PE) (Data S1B), or cerebrospinal fluid (CSF) (Data S1C) using the LiquidBiopsy rare cell isolation system (LIQUIDBIOPSY400A, Zhuhai Sanmed Biotech Ltd.) with capture cocktail kit (biotinylated anti-EpCAM/HER2/Trop2 antibody),^32^ and biotinylated anti-PD-L1 antibody, and streptavidin beads under magnetic field with sheath buffer in a microfluidic chip. After on-chip IF phenotyping (CK, PD-L1, CD45, DAPI) and enumeration under a Leica fluorescence microscope (Leica DM6000B, Germany), the chips were washed, and the recovered cells were resuspended in freezing medium (RPMI-1640 + 10% DMSO) and stored at −80°C. The corresponding patients had completed PD-1/PD-L1 blockade therapy and had documented clinical responses according RECIST v1.1 guidelines in The Fifth Affiliated Hospital of Sun Yat-sen University (Figure S2).

For integrated IF/FISH analysis, cryopreserved CTCs were rapidly thawed at 37°C, washed twice in cold binding buffer (PBS +10% FBS), and deposited onto poly-*l*-lysine–coated slides. Endogenous peroxidase activity was quenched with 0.5% H_2_O_2_ in PBS for 15 min at 37°C. Sequential tyramide-based IF was performed as follows: (i) mouse anti-CK antibody (2 μg/mL) followed by TSA-594 (1 μg/mL) for 30 min; (ii) a second peroxidase block (1% H_2_O_2_, 15 min, 37°C), anti-PD-L1 antibody (2 μg/mL) and TSA-488 (1 μg/mL) for 30 min; (iii) a third peroxidase block (1% H_2_O_2_, 15 min, 37°C), mouse anti-CD45 antibody (5 μg/ml) and TSA-568 (1 μg/mL) for 30 min; (iv) a forth peroxidase block (1% H_2_O_2_, 15 min, 37°C), mouse anti-vimentin antibody (5 μg/ml) and TSA-440 (1 μg/mL) (Cat: 44900; AAT bioquest, USA) for 30 min. Slides were mounted in PBS–DAPI and imaged on a Leica DMB6000 microscope (10× air objective).

After imaging, coverslips were removed, and slides were processed for FISH targeting chromosome 10. Briefly, slides were washed by using 2× SSC at 74°C for 2 min and digested by using 0.1% protease for 5 min at 37°C, then dehydrated through an ethanol series (70%, 85%, 100%, 1 min each). The chromosome 10 probe set (Empire Genomics) was denatured for 5 min at 78°C, applied to the slides, and hybridized overnight at 39°C in a humidified chamber. Post-hybridization washes were performed with 0.4× SSC at 74°C for 2 min and 2× SSC at 37°C for 3 min. Nuclei were counterstained with DAPI. Fluorescence signals were acquired on a Bioview Duet imaging system equipped with a 60× oil-immersion objective.

### Quantification and statistical analysis

Statistical analyses were performed using GraphPad Prism 8.2.0 and SPSS Statistics 25 (IBM). Differences in CTC/CTEC counts among various subgroups were evaluated using Student’s *t* test. The receiver operating characteristic (ROC) curve analysis was conducted to determine the optimal cutoff value of CK^−^/PD-L1^+^ cells associated with ORR. Cutoff value was deemed reliable when the Area under the curve (AUC) exceeded 0.8.^34^ The correlation of the dynamic changes in PD-L1+ CTECs to tumor biomarkers, PD-L1^+^ WBCs to C-reactive protein (CRP) levels was assessed using Spearman’s rank correlation coefficient (Spearman’s rho) in SPSS Statistics 25 (IBM).

### Additional resources

The clinical samples used in this study are approved by the ethics committee of The Fifth Affiliated Hospital, Sun Yat-sen University (ethics review board The Fifth Affiliated Hospital Sun Yat-sen University approval number: K278-1, clinical registration number: ChiCTR2400080132), and all enrolled patients sign the informed consents.

## Supplemental information

Document S1. Figures S1–S3 and Tables S1–S5

Data S1. CTCs isolated from blood, pleural/peritoneal effusion (PE), and cerebrospinal fluid (CSF), respectively, relative to figure 4 (A) Description of CTCs isolation from blood; (B) description of CTCs isolation from PE; (C) description of CTCs isolation from CSF.
