# Supplementary material for: Analyzed PD-L1-positive subpopulations by dual-labeling TSA-IF-FISH predicts immunotherapy efficacy in advanced lung cancer
Source: iScience. 2025 Dec 6;29(1):114357. doi: 10.1016/j.isci.2025.114357 (PMC12799786; doi:10.1016/j.isci.2025.114357)
Supplement: Document S1. Figures S1–S3 and Tables S1–S5 [file mmc1.pdf]

iScience, Volume *29*

Supplemental information

Analyzed PD-L1-positive subpopulations by dual-labeling TSA-IF-FISH predicts immunotherapy efficacy in advanced lung cancer

Lin Chen, Zhonglin Yang, Yue Lu, Shan Li, Dongjiang Tang, and Lei Zhang

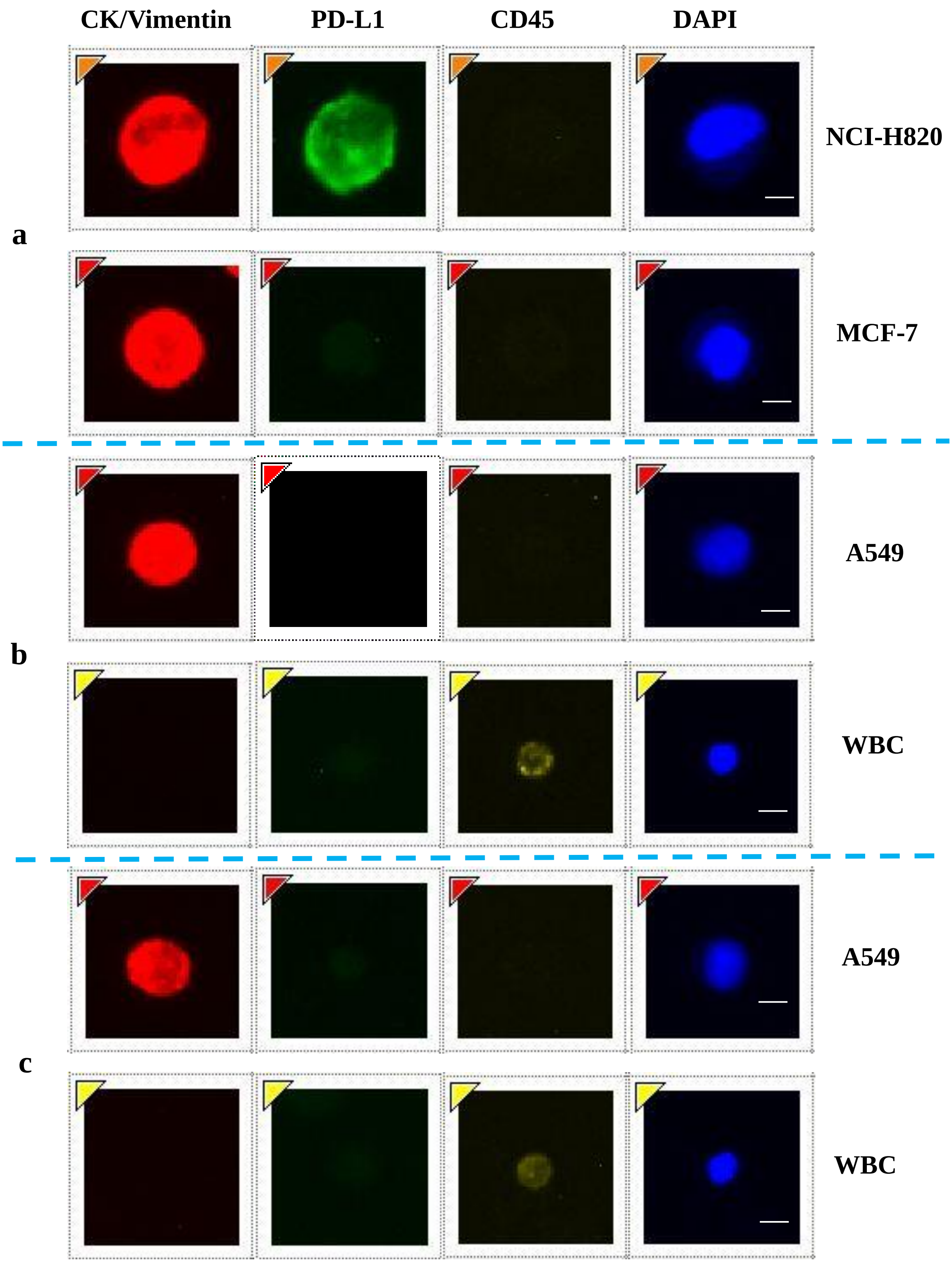

Supplementary Figure S1. Specificity validation of the immunofluorescence (IF) assay for detecting CK, Vimentin, and PD-L1. a: Evaluation of PD-L1 detection specificity. NCI-H820 (PD-L1-positive) and MCF-7 (PD-L1-negative) cell lines were incubated with a cocktail of fluorescent antibodies (CK-iFluor647, PD-L1-iFluor488, CD45-iFluor568). b: Evaluation of CK detection specificity. A549 (CK positive) and white blood cells (WBC, CK negative) cells were incubated with a cocktail of fluorescent antibodies (CK-iFluor647, PD-L1-iFluor488, CD45-iFluor568). c: Evaluation of Vimentin detection specificity. A549 cells (Vimentin-positive)

and white blood cells (Vimentin-negative) were stained with antibody cocktail of CK-iFluor647, PD-L1-iFluor488, and CD45-iFluor568. All samples were imaged using a Leica fluorescence microscope. The observed IF phenotypes were as expected: NCI-H820 (CK+/PD-L1+/CD45-), MCF-7 (CK+/PD-L1-/CD45-), A549 (CK+/Vimentin+/CD45-), and WBCs (CK-/Vimentin-/CD45+). Bars: 8μm.

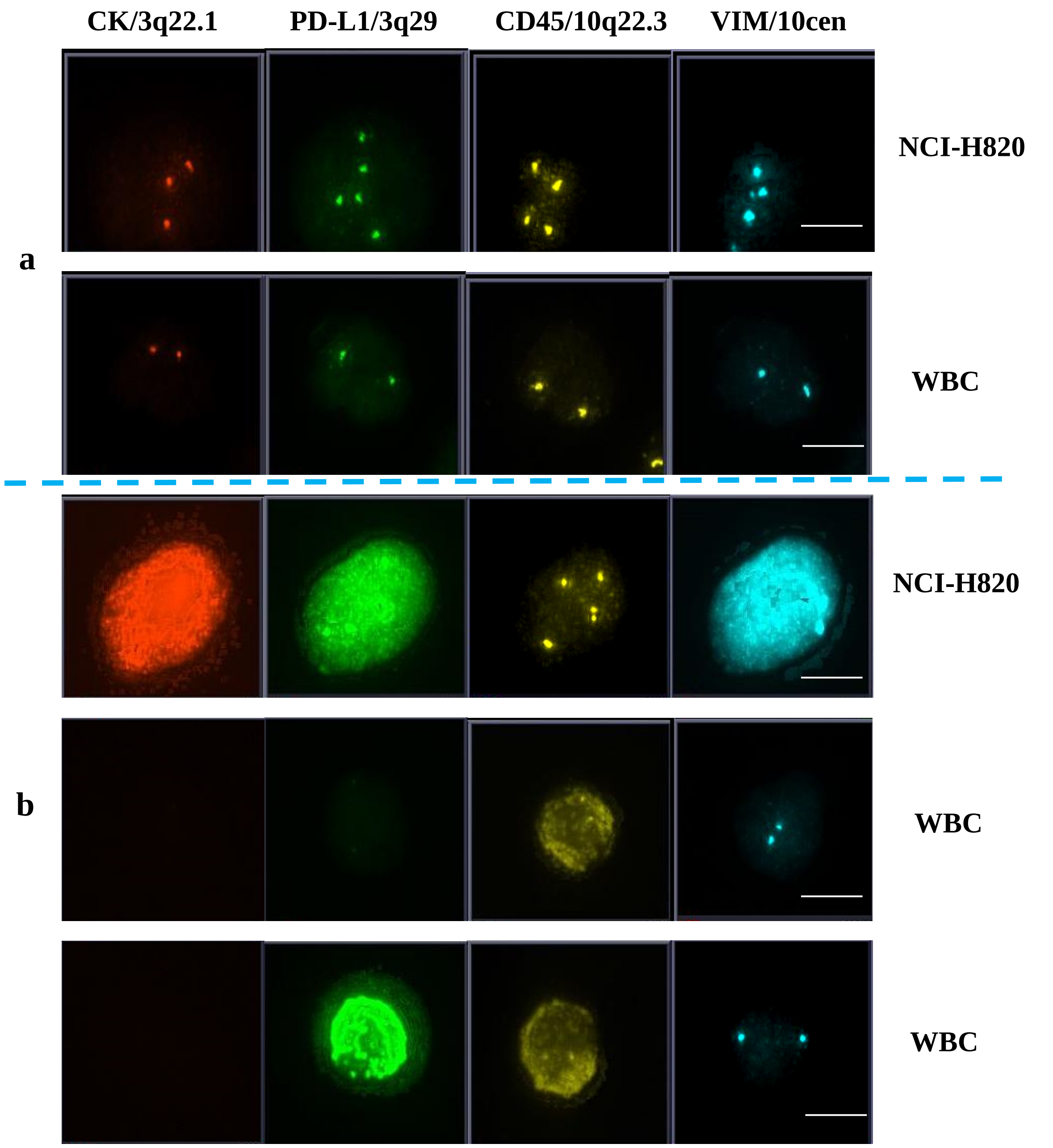

Supplementary Figure S2. Specificity validation of fluorescence in situ hybridization (FISH) and dual TSA-IF/FISH assays. a: Validation of FISH probe specificity. The FISH assay was performed on NCI-H820 cells (with known chromosomal aberrations) and normal white blood cells (WBCs) using a probe cocktail (Red/3q22.1, Green/3q29, Gold/10q22.3, Aqua/10cen). As expected, NCI-H820 tumor cells exhibited more than two hybridization signals for each probe, indicating chromosomal abnormalities, whereas WBCs consistently showed two signals per probe, confirming a disomic (normal) genotype. **b: Validation of the sequential dual TSA-IF/FISH assay.** The same cell types (NCI-H820 and WBCs) were first subjected to tyramide signal amplification (TSA)-based immunofluorescence (IF). Cells were sequentially stained with HRP-conjugated antibodies: incubation with anti-CK followed by TSA-594, anti-PD-L1 followed by TSA-488, anti-CD45 followed by TSA-568, and anti-Vimentin (VIM) followed by TSA-440. Following TSA-IF, FISH was performed using probes for Gold/10q22.3 and Aqua/10cen. The results confirmed the expected phenotypes: NCI-H820 cells were CK+/PD-L1+/CD45-/Vimentin+

with aberrant chromosome 10 signals, while WBCs were either CK-/PD-L1-/CD45+/Vimentin- or CK-/PD-L1+/CD45+/Vimentin- and displayed two normal signals for chromosome 10. Bars: 8μm.

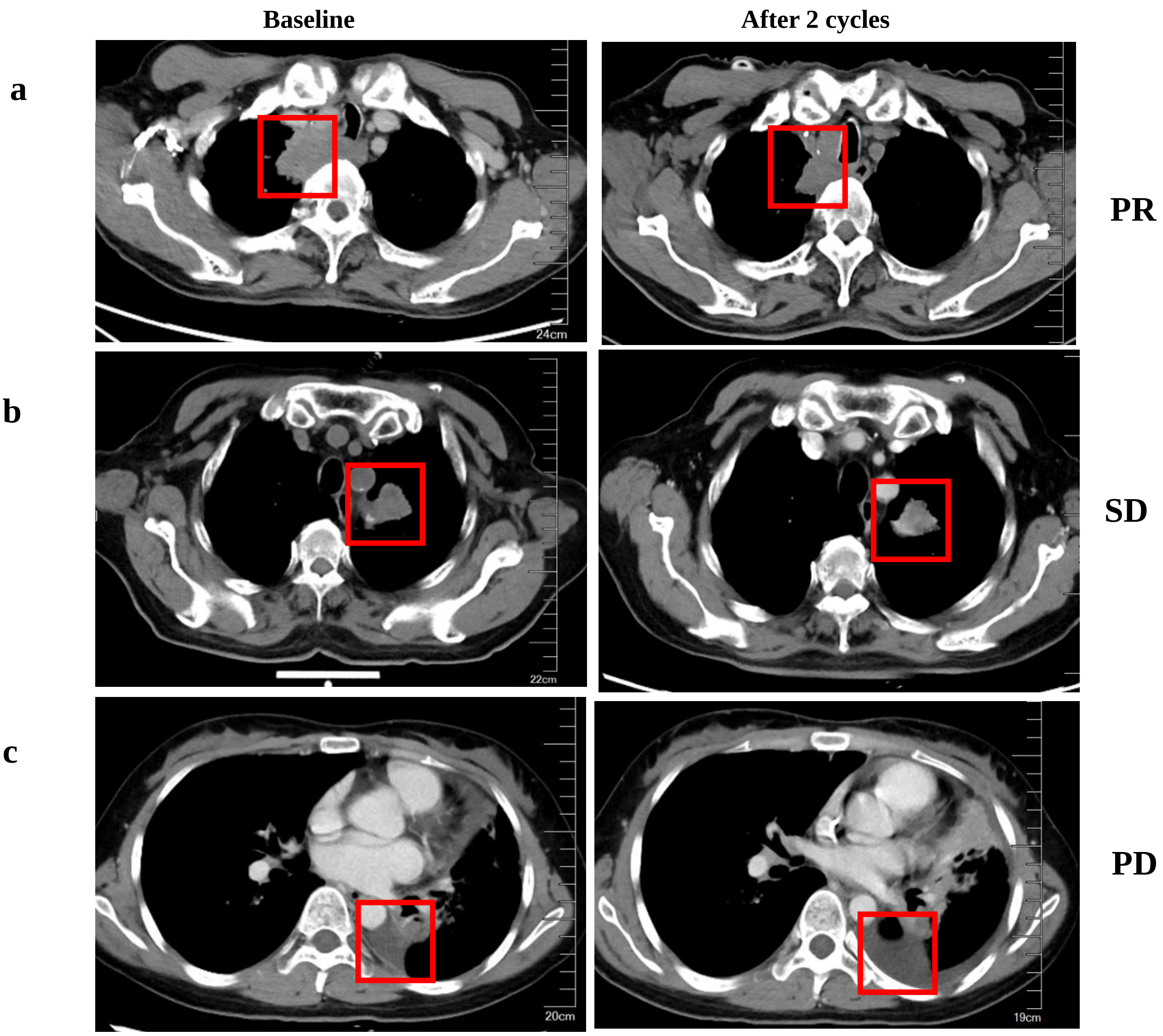

Supplementary Figure S3. Clinical benefit from treatment based on CT imaging. a: A patient acquired partial response (PR) after combination therapy of Paclitaxel, Cisplatin, Tislelizumab for 2 cycles; b: A patient achieved SD under combination therapy of Albumin paclitaxel, Sintilimab after 2 cycles. c: A patient got PD after receiving 2 cycles of combination therapy of Gemcitabine, Carboplatin, and Tislelizumab. Tumor tissue was outlined in red.

Supplementary Table 1: Assay accuracy assessed based on cell recovery rates

Expected cells	Test 1 (%)	Test 2 (%)	Test 3 (%)	Test 4 (%)
5	80	60	60	100
25	84	88	84	92
100	85	93	90	96
500	94	97	92	90
1000	90	94	93	90
Average	86.6	86.4	83.8	93.6
SD	5.46	15.11	13.75	4.34

Performance of the enrichment workflow was assessed by determining the recovery rate of spiked NCI-H820 cells. The tumor cells were spiked into 5 mL of healthy donor blood at known concentrations (5, 25, 100, 500, 1000). After PBMC isolation by Ficoll gradient centrifugation, the cells were subjected to immunostaining with biotinylated capture cocktails and fluorescent anti-CK/PD-L1/CD45 antibodies, followed by magnetic enrichment via the LiquidBiopsy™ system. The recovery rate for each concentration was calculated by dividing the number of cells identified by fluorescence microscopy (Leica) by the number of cells originally spiked.

Supplementary Table 2: Assay precision evaluated on recovered cells

Test	Cells
1	80
2	83
3	88
4	92
5	85
6	93
7	89
8	86
9	90
10	87
Average	87.3
SD	4.0
CV	4.5%

To evaluate technical precision, multiple replicate samples (n = 10) containing 100 spiked NCI-H820 cells in 5 mL of healthy donor blood were processed independently through the complete workflow (PBMC isolation, immunostaining, and magnetic enrichment). The repeatability of the assay was quantified by calculating the standard deviation and coefficient of variation (CV) of the cell recovery rates across these replicates.

Supplementary Table 3: CTCs/CTECs assessed on healthy donors and benign pulmonary nodule

Healthy donor	PD-L1$^+$ CTC	PD-L1$^+$ CTEC	PD-L1$^+$ WBC
HD1	0	0	2
HD2	0	0	4
HD3	0	0	9
HD4	0	0	2
HD5	0	0	4
HD6	0	0	8
HD7	0	0	3
HD8	0	0	3
HD9	0	0	4
HD10	0	0	5
Benign pulmonary nodule			
B1	0	0	7
B2	0	0	3
B3	0	0	4
B4	0	0	6
B5	0	0	4
B6	0	0	5
B7	0	0	5
B8	0	0	4
B9	0	0	4
B10	0	0	3
Lung cancer			
P1	0	2	8
P2 (Immune pneumonia)	3	3	90
P3	0	0	8
P4	4	0	5
P5	3	0	7
P6 (Immune pneumonia)	2	10	113
P7	2	0	9
P8	0	0	5
P9	1	0	6
P10	0	2	5

To validate the specificity of our assay, we analyzed samples from 10 healthy volunteers and 10 patients with benign pulmonary nodules, who served as control groups, and compared them with samples from 10 lung cancer patients. No PD-L1$^+$ CTC or CTECs were detected in the control groups. Furthermore, the level of PD-L1$^+$ WBCs was elevated in the lung cancer cohort with immune pneumonia in P2 and P6.

Supplementary table 4: Results of tPD-L1 and CTC PD-L1 in 24 lung cancer patients.

Patients ID	tPD-L1	CTC PD-L1
1	0%	0
2	10%	3
3	0%	0
4	0%	4
5	20%	3
6	0%	2
7	60%	2
8	90%	0
9	0%	1
10	95%	0
11	10%	9
12	90%	3
13	0%	1
14	0%	4
15	20%	6
16	0%	6
17	70%	0
18	0%	2
19	0%	4
20	5%	6
21	10%	0
22	0%	0
23	5%	0
24	15%	0

Supplementary Table 5: Correlation analysis of PD-L1⁺ CTC to tumor tissue PD-L1.

Variable 1	Variable 2	Spearman's rho	p-value
tPD-L1	CTC PD-L1	-0.138	0.521

* Significant relevance at p<0.05
