# Supplementary material for: Analyzed PD-L1-positive subpopulations by dual-labeling TSA-IF-FISH predicts immunotherapy efficacy in advanced lung cancer
Source: iScience. 2025 Dec 6;29(1):114357. doi: 10.1016/j.isci.2025.114357 (PMC12799786; doi:10.1016/j.isci.2025.114357)
Supplement: Data S1. CTCs isolated from blood, pleural/peritoneal effusion (PE), and cerebrospinal fluid (CSF), respectively, relative to figure 4 — (A) Description of CTCs isolation from blood; (B) description of CTCs isolation from PE; (C) description of CTCs isolation from CSF. [file mmc2.pdf]

DATA S1: CTCs isolated from blood, pleural/peritoneal effusion (PE), and Cerebrospinal fluid (CSF), respectively. Relative to figure 4. A) Description of CTCs isolation from blood; B) Description of CTCs isolation from PE; C) Description of CTCs isolation from CSF.

DATA S1 A: CTCs isolated from blood.

The procedure for isolating and analyzing circulating tumor cells (CTCs) was as follows:

- **Blood Collection and PBMC Isolation:** 5-10 mL of blood was drawn into Cwbio tubes （Cat: CW2815M; CWBIO, Jiangsu, China). PBMCs were isolated via Ficoll-Paque (Cat: P8610; Solarbio, Beijing, China) density gradient centrifugation (1000 × g, 10 min).
- **Cell Fixation:** Cells were fixed with 1% paraformaldehyde (PFA) (Cat：P1110；Solarbio，Beijing; China） at room temperature for 60 min.
- **Immunostaining:** Fixed cells were incubated overnight at 2-8°C with 20μL cocktail of biotinylated capture antibodies (against HER2, Trop2, EpCAM, and PD-L1) and fluorescent detection antibodies (4μg/mL CK-iFluor 647, 4μg/mL PD-L1-iFluor 488, 5μg/mL CD45-iFluor 568).
- **Magnetic Enrichment:** Cells were labeled with 30μL streptavidin-magnetic beads (60 min, 2-8°C) and enriched using the LiquidBiopsy™ system (LIQUIDBIOPSY400A, Zhuhai Sanmed Biotech Ltd.).
- **Analysis and Storage:** Enriched cells were stained with 5μL DAPI, identified and counted via Leica fluorescence microscopy (Leica DM6000B, Germany), then collected by centrifugation and cryopreserved in RPMI-1640/10% DMSO at −80 °C.

DATA S1 B: CTCs isolated from pleural/peritoneal effusion (PE)

- **Sample Collection and CTC Isolation:** 5-10 mL of PE was drawn into Cwbio tubes （Cat: CW2815M; CWBIO, Jiangsu, China). PBMCs were isolated via Ficoll-Paque (Cat: P8610; Solarbio, Beijing, China) density gradient centrifugation (1000 × g, 10 min).
- **Cell Fixation:** Cells were fixed with 1% paraformaldehyde (PFA) (Cat：P1110；Solarbio，Beijing; China）at room temperature for 60 min.
- **Immunostaining:** Fixed cells were incubated overnight at 2-8°C with 20μL cocktail of biotinylated capture antibodies (against HER2, Trop2, EpCAM, and PD-L1) and fluorescent detection antibodies (4μg/mL CK-iFluor 647, 4μg/mL PD-L1-iFluor 488, 5μg/mL CD45-iFluor 568).
- **Magnetic Enrichment:** Cells were labeled with 30μL streptavidin-magnetic beads (60 min, 2-8°C) and enriched using the LiquidBiopsy^{TM} system (LIQUIDBIOPSY400A, Zhuhai Sanmed Biotech Ltd.).
- **Analysis and Storage:** Enriched cells were stained with 5μL DAPI, identified and counted via Leica fluorescence microscopy (Leica DM6000B, Germany), then collected by centrifugation and cryopreserved in RPMI-1640/10% DMSO at −80 °C.

DATA S1 C: CTCs isolated from Cerebrospinal fluid (CSF)

- **Sample collection and CTC enrichment:** CSF samples (5-10 mL) were collected using Cwbio collection tubes. The samples were transferred to 15 mL centrifuge tubes and centrifuged at 1000 × g for 5 minutes to pellet the cells. The cell pellet was then fixed with 1% PFA at room temperature for 30 minutes, followed by two washes with binding buffer.
- **Immunostaining:** Fixed cells were incubated overnight at 2-8°C with 20μL cocktail of biotinylated capture antibodies (against HER2, Trop2, EpCAM, and PD-L1) and fluorescent detection antibodies (4μg/mL CK-iFluor 647, 4μg/mL PD-L1-iFluor 488, 5μg/mL CD45-iFluor 568).
- **Magnetic Enrichment:** Cells were labeled with 30μL streptavidin-magnetic beads (60 min, 2-8°C) and enriched using the LiquidBiopsy™ system (LIQUIDBIOPSY400A, Zhuhai Sanmed Biotech Ltd.).
- **Analysis and Storage:** Enriched cells were stained with 5μL DAPI, identified and counted via Leica fluorescence microscopy (Leica DM6000B, Germany), then collected by centrifugation and cryopreserved in RPMI-1640/10% DMSO at −80 °C.
